# Supplementary material for: The N-Glycosylation of Total Plasma Proteins and IgG in Atrial Fibrillation
Source: Biomolecules. 2023 Mar 28;13(4):605. doi: 10.3390/biom13040605 (PMC10135591; doi:10.3390/biom13040605)
Supplement: Supplementary file 1 [file biomolecules-13-00605-s001.zip › biomolecules-2268750-supplementary.pdf]

Supplementary material for

N-glycosylation of total plasma proteins and IgG in atrial fibrillation

Branimir Plavša[1], Janko Szavits Nossan[2], Aleksandar Blivajs[3], Borna Rapčan[1], Barbara Radovani[4], Lovorka Đerek[3], Diana Rudan[3], Gordan Lauc [1,5], Ivan Gudelj[4,5]

Affiliations:

1. Faculty of Pharmacy and Biochemistry, University of Zagreb, Zagreb, Croatia
2. Magdalena Clinic for Cardiovascular Disease, Krapinske Toplice, Croatia
3. Department of Cardiology, University Hospital Dubrava, Zagreb, Croatia
4. Department of Biotechnology, University of Rijeka, Rijeka, Croatia
5. Genos Glycoscience Research Laboratory, Zagreb, Croatia

Table S1. Formulas used for the calculation of derived traits for plasma and IgG.

Derived trait	Formula
**Plasma**	
Low branching (mono- and biantennary glycans) (**LB**)	GP1+GP2+GP3+GP4+GP5+GP6+GP8+GP9+GP10+GP11+GP12+GP13+GP14+GP15+GP16+GP17+GP18+GP20+GP21+GP22+GP23
High branching (tri- and tetraantennary glycans) (**HB**)	GP24+GP25+GP26+GP27+GP28+GP29+GP30+GP31+GP32+GP33+GP34+GP35+GP36+GP37+GP38+GP39
Agalactosylation (**G0**)	GP1+0.5xGP2
Monogalactosylation (**G1**)	GP3+GP4+GP5+GP6+GP13
Digalactosylation (**G2**)	GP8+GP9+GP10+GP11+0.5xGP12+GP14+GP15+GP16+GP17+GP18+GP20+GP21+GP22+GP23
Trigalactosylation (**G3**)	GP24+GP25+GP26+GP27+GP28+GP29+GP30+GP31+GP32+GP33+GP34+GP35
Tetragalactosylation (**G4**)	GP36+GP37+GP38+GP39
Neutral glycans (**S0**)	GP1+0.5xGP2+GP3+GP4+GP5+GP6+GP8+GP9+GP10+GP11
Monosialylation (**S1**)	0.5xGP12+GP13+GP14+GP15+GP16+GP17
Disialylation (**S2**)	GP18+GP20+GP21+GP22+GP23+GP24+GP25+GP26+GP27
Trisialylation (**S3**)	GP28+GP29+GP30+GP31+GP32+GP33+GP34+GP35+GP36
Tetrasialylation (**S4**)	GP37+GP38+GP39
Bisecting GlcNAc (**B**)	0.5xGP2+GP3+GP6+GP9+GP11+GP15+GP17+ GP23
Oligomannose glycans (**M**)	0.5xGP2+GP7+0.5xGP12+GP19
Core fucosylation (**CF**)	GP1+0.5xGP2+GP4+GP5+GP6+GP10+GP11+GP13+GP16+GP17+GP22+GP23+GP31+GP34+GP35
Antennary fucosylation (**AF**)	GP27+GP33+GP35+GP39
**IgG**	
Agalactosylation (**G0**)	IGP1+IGP2+IGP3+IGP4+IGP6
Monogalactosylation (**G1**)	IGP7+IGP8+IGP9+IGP10+IGP11
Digalactosylation (**G2**)	IGP12+IGP13+IGP14+IGP15
Sialylation (**S**)	IGP16+IGP17+IGP18+IGP19+IGP20+IGP21+IGP22+IGP23+IGP24
Bisecting GlcNAc (**B**)	IGP3+IGP6+IGP10+IGP11+IGP13+IGP15+IGP19+IGP22+IGP24
Core fucosylation (**CF**)	IGP1+IGP4+IGP6+IGP8+IGP9+IGP10+IGP11+IGP14+IGP15+IGP16+IGP18+IGP19+IGP23+IGP24

Table S2. Differences in total plasma protein N-glycans between controls and AF patients corrected for age and sex. Significant associations are given in bold.

Glycan trait	b-coefficient (95% confidence interval)*	P-value	Adjusted p-value[#]
GP01	-0.105 (-0.248, 0.037)	0.1465	0.379
GP02	-0.094 (-0.163, -0.024)	0.0083	0.095
GP03	-0.145 (-0.273, -0.017)	0.0265	0.252
GP04	-0.055 (-0.177, 0.067)	0.3784	0.674
GP05	0.001 (-0.145, 0.146)	0.9925	0.993
GP06	-0.12 (-0.205, -0.035)	0.0057	0.081
GP07	-0.016 (-0.072, 0.04)	0.5771	0.82
GP08	-0.005 (-0.048, 0.039)	0.8363	0.941
GP09	-0.022 (-0.097, 0.053)	0.5597	0.82
GP10	0.009 (-0.083, 0.102)	0.8422	0.941
GP11	-0.036 (-0.124, 0.052)	0.4179	0.722
GP12	-0.005 (-0.051, 0.041)	0.8351	0.941
GP13	0.021 (-0.08, 0.122)	0.6823	0.884
GP14	0.009 (-0.027, 0.045)	0.6128	0.832
GP15	0.001 (-0.064, 0.066)	0.9819	0.993
GP16	0.038 (-0.013, 0.09)	0.1457	0.379
GP17	-0.001 (-0.094, 0.092)	0.9815	0.993
GP18	0.018 (-0.04, 0.076)	0.5416	0.82
**GP19**	**0.075 (0.036, 0.114)**	**0.0002**	**0.004**
GP20	-0.004 (-0.044, 0.035)	0.8265	0.941
GP21	-0.008 (-0.052, 0.035)	0.7088	0.898
GP22	0.046 (-0.015, 0.107)	0.136	0.379
GP23	0.043 (-0.048, 0.135)	0.352	0.669
GP24	0.021 (-0.056, 0.097)	0.5896	0.82
GP25	-0.003 (-0.055, 0.049)	0.8981	0.984
GP26	-0.031 (-0.088, 0.026)	0.2863	0.583
GP27	-0.102 (-0.209, 0.005)	0.0616	0.322
GP28	0.039 (-0.047, 0.125)	0.3712	0.674
GP29	0.077 (-0.002, 0.155)	0.0567	0.322
GP30	0.021 (-0.054, 0.096)	0.5731	0.82
GP31	0.001 (-0.087, 0.089)	0.9816	0.993
GP32	-0.067 (-0.141, 0.006)	0.0734	0.322
GP33	-0.08 (-0.185, 0.026)	0.1391	0.379
GP34	-0.041 (-0.104, 0.021)	0.1937	0.46
GP35	-0.092 (-0.193, 0.008)	0.072	0.322
GP36	-0.044 (-0.1, 0.011)	0.1192	0.379
GP37	0.013 (-0.075, 0.1)	0.7727	0.937
GP38	-0.021 (-0.081, 0.039)	0.4911	0.8
GP39	-0.103 (-0.201, -0.004)	0.0413	0.322
LB	0.006 (-0.004, 0.016)	0.2152	0.486

HB	-0.03 (-0.074, 0.014)	0.1766	0.438
G0	-0.099 (-0.223, 0.024)	0.113	0.379
G1	-0.042 (-0.152, 0.068)	0.4551	0.763
G2	0.016 (-0.001, 0.033)	0.0723	0.322
G3	-0.028 (-0.074, 0.017)	0.2215	0.486
G4	-0.047 (-0.106, 0.012)	0.1182	0.379
S0	-0.046 (-0.139, 0.046)	0.3261	0.641
S1	0.021 (0, 0.042)	0.0482	0.322
S2	0.005 (-0.027, 0.036)	0.7711	0.937
S3	-0.028 (-0.074, 0.018)	0.2337	0.493
S4	-0.047 (-0.11, 0.015)	0.1376	0.379
Bisecting	-0.018 (-0.079, 0.043)	0.5622	0.82
OligoMann	-0.008 (-0.042, 0.025)	0.6344	0.841
CoreFuc	-0.002 (-0.061, 0.058)	0.9515	0.993
AnteFuc	-0.088 (-0.189, 0.013)	0.0881	0.359

*b-coefficient represents the natural logarithm of the relative change in N-glycan traits between groups corrected for age and sex differences.

# p-value was adjusted using the Li-Ji correction method

Table S3. Differences in IgG N-glycans between controls and AF patients corrected for age and sex. Significant associations are given in bold.

Glycan trait	b-coefficient (95% confidence interval)*	P-value	Adjusted p-value#
IGP01	-0.007 (-0.108, 0.094)	0.8877	0.917
IGP02	-0.132 (-0.303, 0.039)	0.1294	0.313
IGP03	0.01 (-0.056, 0.076)	0.7689	0.824
IGP04	-0.039 (-0.105, 0.027)	0.2445	0.426
**IGP05**	**0.123 (0.042, 0.204)**	**0.003**	**0.021**
**IGP06**	**-0.093 (-0.167, -0.019)**	**0.0134**	**0.05**
IGP07	-0.094 (-0.233, 0.045)	0.1833	0.351
IGP08	0.009 (-0.017, 0.034)	0.4948	0.66
**IGP09**	**0.065 (0.02, 0.109)**	**0.0048**	**0.028**
**IGP10**	**-0.083 (-0.143, -0.024)**	**0.0062**	**0.03**
IGP11	-0.029 (-0.087, 0.029)	0.322	0.495
IGP12	-0.1 (-0.245, 0.046)	0.1774	0.351
IGP13	0.009 (-0.054, 0.072)	0.7715	0.824
IGP14	0.051 (-0.018, 0.119)	0.1447	0.315
IGP15	-0.02 (-0.079, 0.04)	0.52	0.66
IGP16	0.029 (-0.021, 0.08)	0.2498	0.426
IGP17	0.049 (-0.015, 0.114)	0.1337	0.313
IGP18	0.021 (-0.046, 0.089)	0.5338	0.66
IGP19	0.001 (-0.072, 0.074)	0.976	0.976
**IGP20**	**0.221 (0.149, 0.294)**	**$8x10^{-9}$**	**$1x10^{-7}$**
**IGP21**	**0.166 (0.095, 0.237)**	**$6x10^{-6}$**	**$6x10^{-5}$**
IGP22	-0.031 (-0.129, 0.066)	0.5289	0.66
IGP23	0.098 (0.004, 0.192)	0.0418	0.14
IGP24	0.014 (-0.061, 0.088)	0.7174	0.822
G0	-0.05 (-0.111, 0.01)	0.1003	0.303
G1	0.009 (-0.01, 0.027)	0.3543	0.52
G2	0.035 (-0.029, 0.098)	0.2854	0.462
S	0.036 (-0.011, 0.083)	0.1275	0.313
**Bisecting**	**-0.055 (-0.096, -0.014)**	**0.0083**	**0.035**
CoreFuc	-0.001 (-0.006, 0.003)	0.5689	0.677

*b-coefficient represents the natural logarithm of the relative change in N-glycan traits between groups corrected for age and sex differences.

# p-value was adjusted using the Li-Ji correction method

Table S4. Associations of total plasma protein N-glycans with the recurrence of AF after catheter ablation. Model 1 is corrected for age and sex, while model 2 is corrected for age, sex, diabetes, and hypertension. Significant associations are given in bold.

Glycan trait	b-coefficient (95% confidence interval)* - model 1	P-value	Adjusted p-value#	b-coefficient (95% confidence interval)* - model 2	P-value	Adjusted p-value#
GP01	0.117 (-0.049, 0.284)	0.165	0.495	0.117 (-0.049, 0.282)	0.1661	0.39
**GP02**	**0.117 (0.038, 0.196)**	**0.0041**	**0.041**	**0.115 (0.036, 0.194)**	**0.0047**	**0.039**
GP03	0.174 (0.028, 0.32)	0.0198	0.113	0.174 (0.03, 0.318)	0.018	0.093
GP04	0.086 (-0.06, 0.232)	0.2465	0.509	0.096 (-0.047, 0.239)	0.1872	0.39
GP05	0.094 (-0.079, 0.266)	0.2862	0.51	0.104 (-0.066, 0.273)	0.2282	0.438
GP06	0.131 (0.033, 0.228)	0.0091	0.065	0.135 (0.039, 0.232)	0.0065	0.046
**GP07**	**0.094 (0.028, 0.159)**	**0.0053**	**0.043**	**0.096 (0.031, 0.162)**	**0.0039**	**0.037**
GP08	0.054 (0.005, 0.104)	0.0328	0.156	0.056 (0.005, 0.106)	0.03	0.142
**GP09**	**0.123 (0.039, 0.207)**	**0.0043**	**0.041**	**0.127 (0.044, 0.21)**	**0.003**	**0.035**
GP10	0.045 (-0.064, 0.154)	0.421	0.614	0.057 (-0.048, 0.161)	0.2882	0.456
GP11	0.098 (-0.004, 0.201)	0.0602	0.264	0.106 (0.005, 0.207)	0.0403	0.177
GP12	0.042 (-0.007, 0.091)	0.0915	0.373	0.041 (-0.008, 0.09)	0.0996	0.379
GP13	0.073 (-0.046, 0.191)	0.2272	0.509	0.079 (-0.039, 0.197)	0.1855	0.39
GP14	0.017 (-0.026, 0.06)	0.4306	0.614	0.017 (-0.026, 0.06)	0.4357	0.608
**GP15**	**0.12 (0.046, 0.195)**	**0.0017**	**0.025**	**0.121 (0.047, 0.196)**	**0.0016**	**0.023**
GP16	0.024 (-0.036, 0.085)	0.4263	0.614	0.03 (-0.03, 0.09)	0.3298	0.495
GP17	0.088 (-0.019, 0.195)	0.1077	0.403	0.094 (-0.014, 0.201)	0.0866	0.353
GP18	-0.029 (-0.096, 0.038)	0.393	0.614	-0.026 (-0.093, 0.041)	0.4465	0.608
GP19	0.024 (-0.02, 0.068)	0.2774	0.51	0.025 (-0.019, 0.069)	0.2682	0.456
GP20	-0.032 (-0.08, 0.016)	0.1894	0.501	-0.035 (-0.082, 0.012)	0.1453	0.39
GP21	-0.013 (-0.063, 0.037)	0.6181	0.705	-0.014 (-0.064, 0.036)	0.5854	0.668
GP22	-0.041 (-0.113, 0.03)	0.2548	0.509	-0.043 (-0.114, 0.029)	0.2384	0.438
GP23	0.061 (-0.044, 0.166)	0.2536	0.509	0.061 (-0.045, 0.167)	0.2574	0.456
GP24	0.025 (-0.058, 0.108)	0.5463	0.677	0.023 (-0.06, 0.107)	0.5821	0.668
GP25	-0.017 (-0.076, 0.043)	0.5755	0.677	-0.021 (-0.08, 0.038)	0.4858	0.629
GP26	0.002 (-0.062, 0.065)	0.9579	0.958	-0.006 (-0.068, 0.055)	0.8397	0.855
GP27	-0.04 (-0.16, 0.08)	0.5105	0.677	-0.04 (-0.161, 0.081)	0.5144	0.645
GP28	-0.003 (-0.096, 0.089)	0.941	0.958	-0.007 (-0.1, 0.086)	0.8843	0.884
GP29	-0.068 (-0.153, 0.016)	0.113	0.403	-0.068 (-0.153, 0.018)	0.1185	0.389
GP30	-0.013 (-0.094, 0.069)	0.7579	0.785	-0.017 (-0.099, 0.064)	0.6753	0.7
GP31	-0.04 (-0.135, 0.055)	0.405	0.614	-0.049 (-0.142, 0.045)	0.3041	0.469

GP32	-0.019 (-0.101, 0.063)	0.6464	0.722	-0.032 (-0.109, 0.046)	0.422	0.608
GP33	-0.043 (-0.161, 0.074)	0.4676	0.635	-0.045 (-0.163, 0.074)	0.4586	0.608
GP34	-0.036 (-0.104, 0.032)	0.2963	0.512	-0.045 (-0.11, 0.02)	0.1726	0.39
GP35	-0.063 (-0.174, 0.048)	0.2653	0.509	-0.067 (-0.179, 0.044)	0.2325	0.438
GP36	0.017 (-0.044, 0.079)	0.5796	0.677	0.014 (-0.048, 0.075)	0.6665	0.7
GP37	-0.027 (-0.118, 0.065)	0.568	0.677	-0.025 (-0.116, 0.066)	0.5861	0.668
GP38	-0.013 (-0.078, 0.052)	0.6957	0.734	-0.014 (-0.079, 0.05)	0.6623	0.7
GP39	-0.022 (-0.131, 0.087)	0.6923	0.734	-0.023 (-0.133, 0.086)	0.6743	0.7
LB	0.006 (-0.004, 0.017)	0.2103	0.509	0.007 (-0.003, 0.017)	0.1559	0.39
HB	-0.023 (-0.072, 0.026)	0.3627	0.608	-0.027 (-0.076, 0.022)	0.276	0.456
G0	0.113 (-0.031, 0.256)	0.1224	0.41	0.112 (-0.031, 0.254)	0.1241	0.389
G1	0.091 (-0.041, 0.222)	0.1744	0.497	0.099 (-0.029, 0.228)	0.1297	0.389
G2	-0.006 (-0.025, 0.014)	0.5816	0.677	-0.006 (-0.025, 0.014)	0.5732	0.668
G3	-0.023 (-0.074, 0.029)	0.3833	0.614	-0.027 (-0.078, 0.023)	0.2882	0.456
G4	-0.014 (-0.078, 0.051)	0.6811	0.734	-0.015 (-0.08, 0.05)	0.6512	0.7
S0	0.079 (-0.031, 0.189)	0.1566	0.495	0.085 (-0.023, 0.193)	0.1227	0.389
S1	0.027 (0.004, 0.051)	0.023	0.119	0.029 (0.006, 0.053)	0.0149	0.085
S2	-0.025 (-0.062, 0.013)	0.1932	0.501	-0.027 (-0.064, 0.01)	0.1475	0.39
S3	-0.029 (-0.081, 0.023)	0.2681	0.509	-0.034 (-0.085, 0.017)	0.191	0.39
S4	-0.022 (-0.091, 0.047)	0.5345	0.677	-0.022 (-0.091, 0.046)	0.5204	0.645
Bisecting	0.09 (0.02, 0.159)	0.0114	0.073	0.092 (0.023, 0.162)	0.0096	0.061
**OligoMann**	**0.069 (0.03, 0.107)**	**0.0006**	**0.012**	**0.069 (0.03, 0.108)**	**0.0006**	**0.011**
CoreFuc	0.042 (-0.028, 0.112)	0.235	0.509	0.046 (-0.023, 0.116)	0.1918	0.39
AnteFuc	-0.042 (-0.154, 0.07)	0.46	0.635	-0.043 (-0.156, 0.07)	0.4494	0.608

*b-coefficient represents the natural logarithm of the relative change in N-glycan traits between groups corrected for age and sex differences.

# p-value was adjusted using the Li-Ji correction method

Table S5. Differences in IgG N-glycans between pre- and post-catheter ablation corrected for age and sex. Significant associations are given in bold.

Glycan trait	b-coefficient (95% confidence interval)*	P-value	Adjusted p-value#
IGP01	-0.017 (-0.101, 0.067)	0.6881	0.979
IGP02	-0.008 (-0.172, 0.156)	0.9237	0.979
IGP03	-0.036 (-0.093, 0.021)	0.213	0.717
IGP04	0.004 (-0.05, 0.058)	0.8751	0.979
**IGP05**	**-0.108 (-0.166, -0.05)**	**0.0003**	**0.002**
IGP06	0.01 (-0.056, 0.075)	0.7726	0.979
IGP07	-0.013 (-0.15, 0.124)	0.8557	0.979
IGP08	0.006 (-0.016, 0.028)	0.5796	0.979
IGP09	-0.005 (-0.043, 0.033)	0.7949	0.979
IGP10	0.013 (-0.041, 0.067)	0.6262	0.979
IGP11	-0.016 (-0.068, 0.036)	0.5358	0.979
IGP12	-0.014 (-0.153, 0.124)	0.8362	0.979
IGP13	-0.006 (-0.06, 0.048)	0.8324	0.979
IGP14	0.001 (-0.058, 0.06)	0.9681	0.979
IGP15	-0.01 (-0.061, 0.04)	0.6823	0.979
IGP16	-0.003 (-0.045, 0.038)	0.8693	0.979
**IGP17**	**-0.086 (-0.143, -0.029)**	**0.0031**	**0.018**
IGP18	-0.001 (-0.062, 0.059)	0.9642	0.979
IGP19	-0.029 (-0.088, 0.03)	0.3319	0.913
**IGP20**	**-0.19 (-0.249, -0.13)**	**$2x10^{-9}$**	**$2x10^{-8}$**
**IGP21**	**-0.172 (-0.227, -0.118)**	**$2x10^{-9}$**	**$2x10^{-8}$**
IGP22	-0.075 (-0.165, 0.015)	0.1024	0.501
IGP23	-0.025 (-0.11, 0.06)	0.5657	0.979
IGP24	-0.037 (-0.096, 0.023)	0.2235	0.717
G0	0.005 (-0.044, 0.053)	0.851	0.979
G1	0.004 (-0.011, 0.018)	0.632	0.979
G2	-0.001 (-0.056, 0.055)	0.9795	0.979
S	-0.024 (-0.064, 0.016)	0.2378	0.717
Bisecting	-0.001 (-0.039, 0.037)	0.9694	0.979
CoreFuc	0.003 (-0.002, 0.008)	0.1893	0.717

*b-coefficient represents the natural logarithm of the relative change in N-glycan traits between groups corrected for age and sex differences.

# p-value was adjusted using the Li-Ji correction method

Table S6. Associations of IgG N-glycans with the CHA_2DS_2-VASc score corrected for age and sex. Significant associations are given in bold.

Glycan trait	Linear b-coefficient (95% confidence interval)	P-value	Adjusted p-value[#]
IGP01	-0.022 (-0.235, 0.191)	0.8382	0.696
IGP02	0.254 (-0.101, 0.61)	0.1597	0.17
IGP03	0.036 (-0.098, 0.17)	0.5959	0.696
**IGP04**	**0.179 (0.04, 0.319)**	**0.0118**	**0.044**
IGP05	-0.095 (-0.263, 0.074)	0.2678	0.243
**IGP06**	**0.355 (0.201, 0.508)**	**$1x10^{-5}$**	**$1x10^{-4}$**
IGP07	0.044 (-0.254, 0.342)	0.7715	0.797
**IGP08**	**-0.067 (-0.119, -0.015)**	**0.0123**	**0.034**
IGP09	-0.079 (-0.17, 0.012)	0.0873	0.168
IGP10	0.141 (0.019, 0.263)	0.0242	0.062
IGP11	0.102 (-0.019, 0.222)	0.0976	0.174
IGP12	-0.055 (-0.369, 0.26)	0.7325	0.783
IGP13	-0.041 (-0.174, 0.091)	0.5377	0.617
**IGP14**	**-0.213 (-0.352, -0.074)**	**0.0028**	**0.014**
IGP15	-0.052 (-0.177, 0.072)	0.4073	0.504
IGP16	-0.047 (-0.155, 0.062)	0.396	0.504
IGP17	-0.076 (-0.216, 0.065)	0.2879	0.387
**IGP18**	**-0.186 (-0.323, -0.048)**	**0.0086**	**0.029**
IGP19	-0.136 (-0.286, 0.013)	0.0735	0.161
IGP20	-0.171 (-0.326, -0.017)	0.0302	0.071
IGP21	-0.121 (-0.277, 0.035)	0.1262	0.205
IGP22	-0.126 (-0.327, 0.076)	0.2195	0.323
**IGP23**	**-0.275 (-0.475, -0.076)**	**0.007**	**0.027**
IGP24	-0.129 (-0.275, 0.018)	0.0852	0.168
**G0**	**0.214 (0.087, 0.341)**	**0.0011**	**0.011**
G1	-0.031 (-0.069, 0.006)	0.1016	0.174
**G2**	**-0.184 (-0.315, -0.053)**	**0.006**	**0.026**
**S**	**-0.152 (-0.248, -0.055)**	**0.0023**	**0.013**
**Bisecting**	**0.132 (0.049, 0.215)**	**0.0019**	**0.013**
CoreFuc	0.004 (-0.006, 0.014)	0.4579	0.545

[#] p-value was adjusted using the Li-Ji correction method

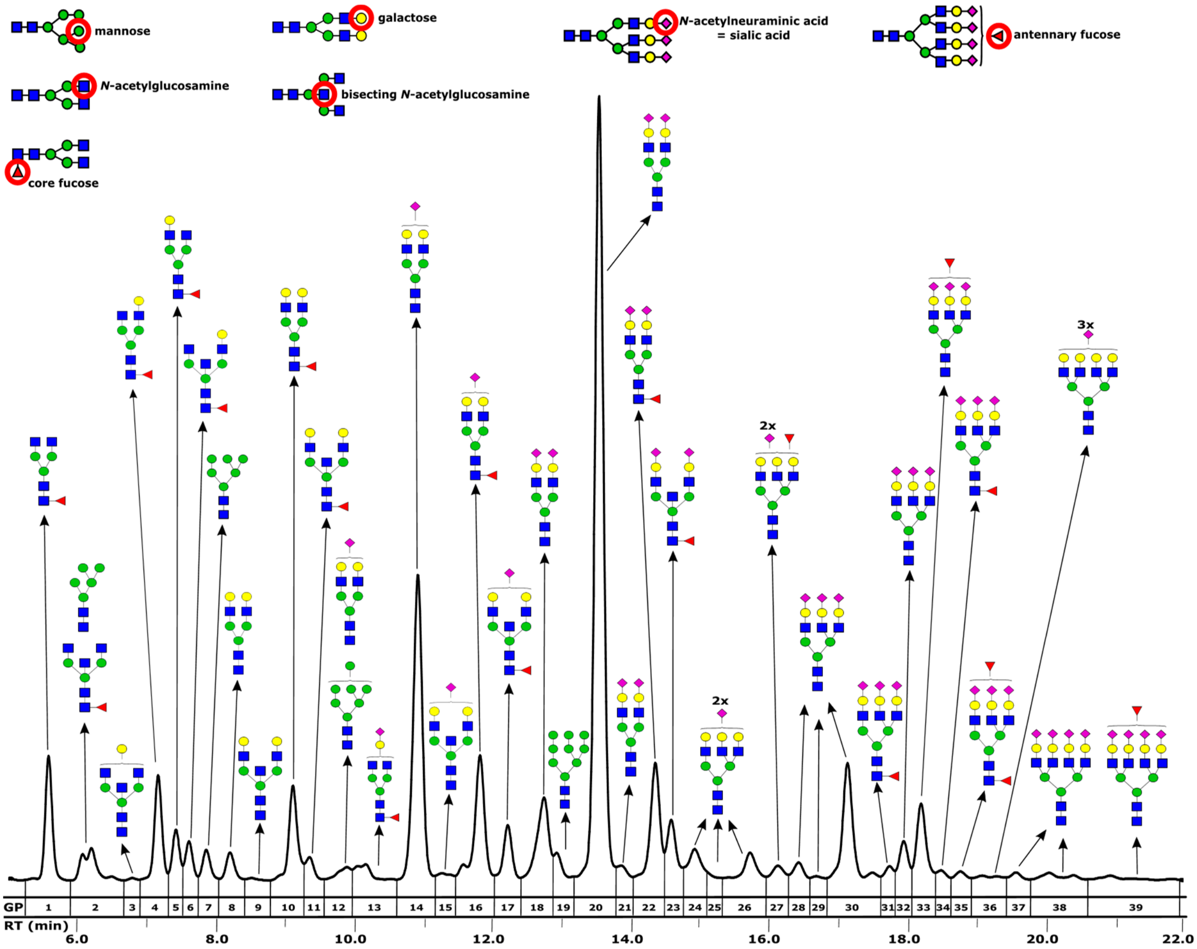

Figure S1. Representative chromatogram of 2-AB labeled total plasma protein N-glycans. Dominant structures in each peak are shown above.

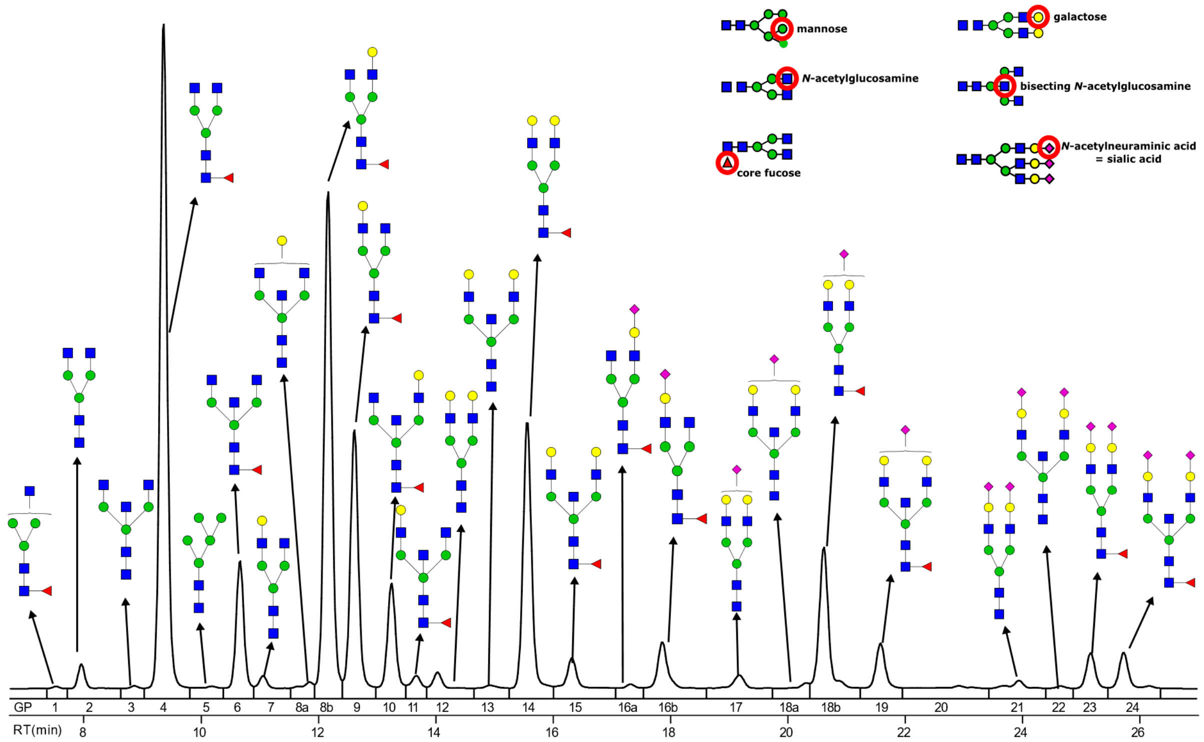

Figure S2. Representative IgG chromatogram with dominant structures in each peak shown.

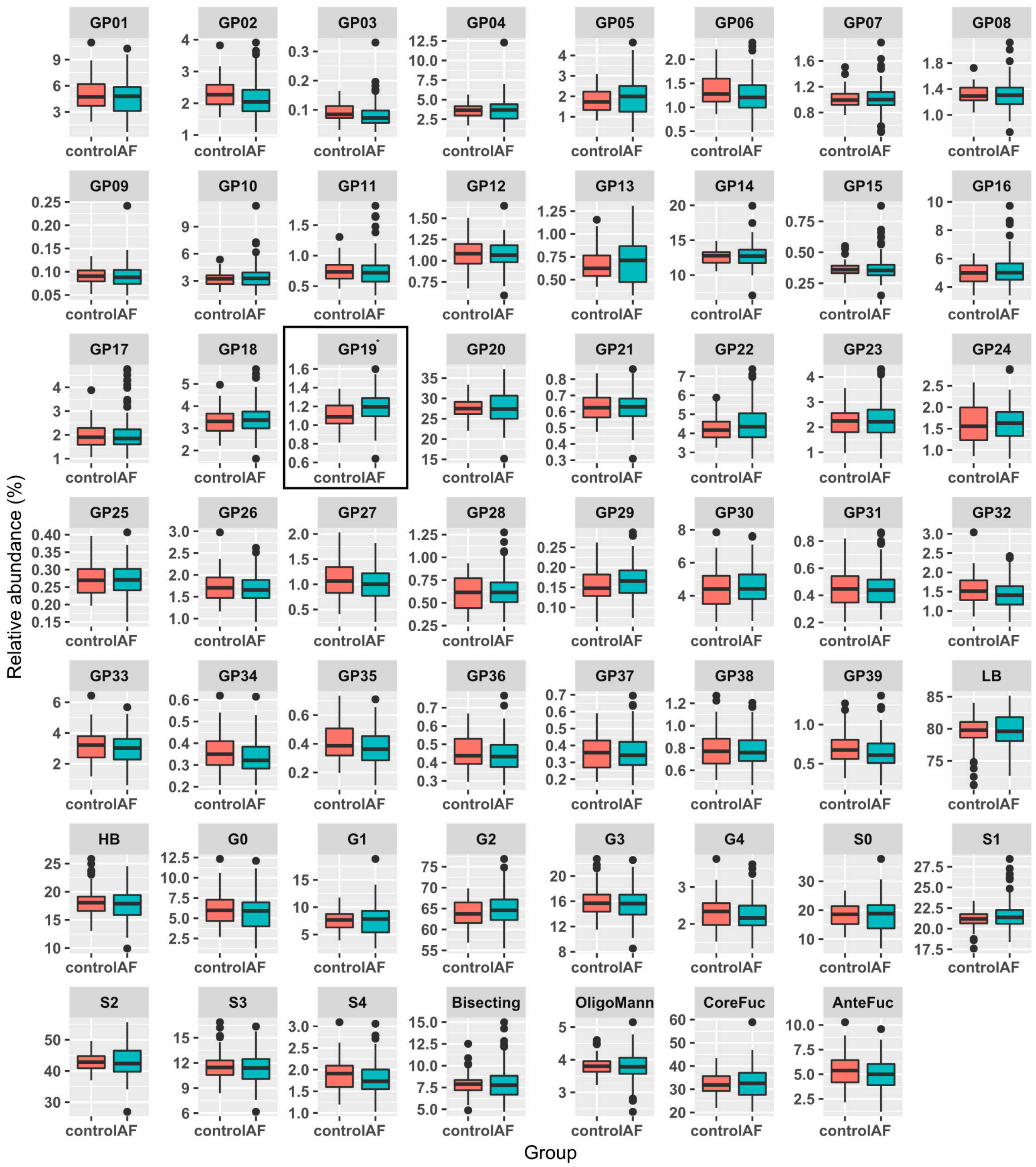

Figure S3. Distribution of total plasma protein N-glycans in AF and control groups. Statistically significant changes in structures are denoted by a box around the plot of the corresponding peak.

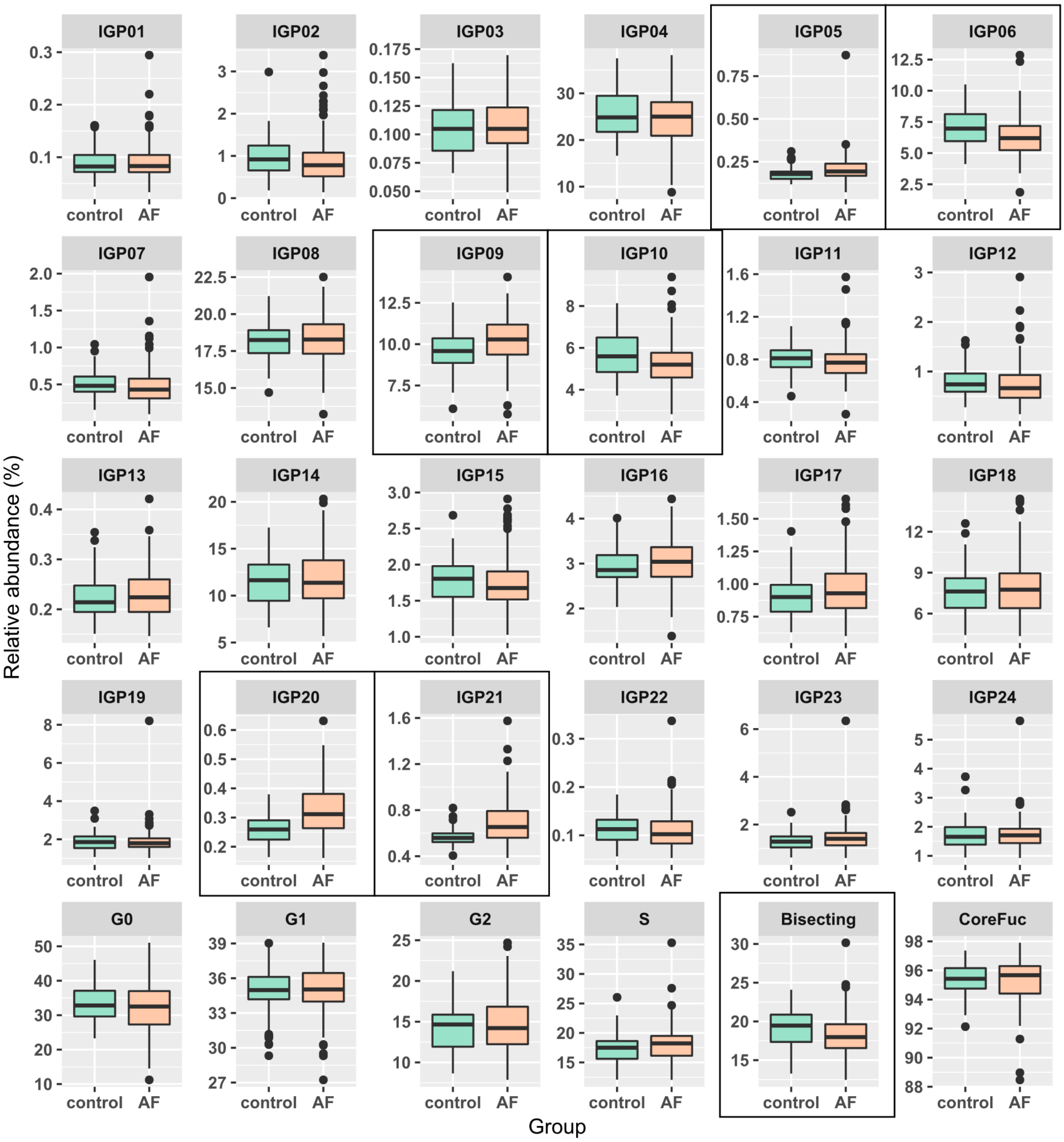

Figure S4. Distribution of IgG N-glycans in AF and control group for all measured IgG N-glycan peaks and derived traits. Structures and traits that are significantly different after adjusting for age and sex are denoted by a box around the corresponding plot.
